# *Plasmodium* sporozoites can invade hepatocytic cells independently of the Ephrin receptor A2

**DOI:** 10.1371/journal.pone.0200032

**Published:** 2018-07-05

**Authors:** Anne-Claire Langlois, Carine Marinach, Giulia Manzoni, Olivier Silvie

**Affiliations:** Sorbonne Université, INSERM, CNRS, Centre d’Immunologie et des Maladies Infectieuses, CIMI-Paris, Paris, France; Johns Hopkins Bloomberg School of Public Health, UNITED STATES

## Abstract

Sporozoite forms of the malaria parasite *Plasmodium* are transmitted by mosquitoes and first infect the liver for an initial round of replication before parasite proliferation in the blood. The molecular mechanisms involved during sporozoite invasion of hepatocytes remain poorly understood. In previous studies, two receptors of the Hepatitis C virus (HCV), the tetraspanin CD81 and the Scavenger Receptor BI (SR-BI), were shown to play an important role during entry of *Plasmodium* sporozoites into hepatocytic cells. In contrast to HCV entry, which requires both CD81 and SR-BI together with additional host factors, CD81 and SR-BI operate independently during malaria liver infection, as sporozoites can use CD81 and/or SR-BI, depending on the *Plasmodium* species, to invade hepatocytes. However, the molecular function of CD81 and SR-BI during parasite entry remains unknown. Another HCV entry factor, the Ephrin receptor A2 (EphA2), was recently reported to play a key role as a host cell entry factor during malaria liver infection. Here, we investigated the contribution of EphA2 during CD81-dependent and SR-BI-dependent sporozoite infection. Using small interfering RNA (siRNA) and antibodies against EphA2, combined with direct detection of parasites by flow cytometry or microscopy, we show that blocking EphA2 has no significant impact on *P*. *yoelii* or *P*. *berghei* host cell infection, irrespective of the entry route. Thus, our findings argue against an important role of EphA2 during malaria liver infection.

## Introduction

Despite some progress in malaria control over the world, 212 million cases still occurred globally in 2016, causing 429 000 deaths, mostly among children under 5 years old in Africa [1]. An effective vaccine would be a powerful tool to finally eradicate the disease. To this end, the liver stage of infection is a suitable target as it is an obligatory gateway for parasite replication. After their inoculation into the skin by infected *Anopheles* mosquitoes, *Plasmodium* sporozoites rapidly migrate to the liver using gliding motility and cell traversal activity. Once in the liver, they first traverse hepatocytes before invading them and developing into exo-erythocytic forms (EEFs), surrounded by a parasitophorous vacuole membrane (PVM). Then, they differentiate into thousands of merozoites that will invade red blood cells and provoke the symptomatic phase of the disease.

Host cell invasion is a complex mechanism that remains poorly understood at the molecular level. Previous studies showed that *Plasmodium* sporozoites share a common set of host entry factors with the hepatotropic Hepatitis C Virus (HCV). HCV entry involves several sequential steps with initial attachment to the host cell surface followed by receptor-dependent intake and clathrin-mediated endocytosis [2]. Liver heparan sulfated proteoglycans (HSPGs) mediate HCV attachment [3,4]. Four hepatocyte membrane receptors play a critical role in the post-attachment steps of invasion, the scavenger receptor type B class I (SR-BI) [5], the tetraspanin CD81 [6] and the tight junction proteins Claudin-1 (CLDN1) [7] and Occludin (OCLN) [8,9]. Similarly to HCV, *Plasmodium* sporozoites attach to HSPGs [10] and exploit CD81 and SR-BI for subsequent invasion [11–13]. However, in contrast with HCV that requires both SR-BI and CD81 for entry, *Plasmodium* sporozoites invade liver cells using either CD81 or SRB1, depending on the *Plasmodium* species [14,15]. Indeed, we have shown that CD81 is essential for *P*. *falciparum* and *P*. *yoelii* sporozoite invasion [13], and facultative for *P*. *berghei* [13,16], which can enter cells via a SR-BI-dependent route in the absence of CD81 [15]. Furthermore, SR-BI (but not CD81) is important for *P*. *vivax* sporozoite infection [15].

Recently, Kaushansky *et al*. reported that host cell susceptibility to *Plasmodium* sporozoite infection correlates with the levels of expression of Ephrin receptor A2 (EphA2), and proposed that EphA2 is an important host receptor for sporozoite invasion [17]. EphA2 is a tyrosine kinase receptor composed of a single kinase intracellular domain, an extracellular region containing a Cys-rich domain and two fibronectin type III repeats. Ephrin receptors are involved in intercellular signaling in metazoans, *via* the binding of ephrin ligands anchored in the membrane of adjacent cells. Interestingly, EphA2 and the Epidermal Growth Factor Receptor (EGFR) are also implicated during HCV entry, where they act by regulating CD81-Claudin-1 co-receptor associations and viral glycoprotein-dependent membrane fusion [18]. Here, we investigated the functional relations between EphA2 and CD81-dependent and independent pathways during *Plasmodium* sporozoite invasion. Since we have shown that *Plasmodium* sporozoites use distinct host entry pathways depending on the parasite species, we explored the implication of EphA2 using different hepatocytic cell types infected with *P*. *yoelii* or *P*. *berghei* sporozoites.

## Materials and methods

### Ethics statement

All animal work was conducted in strict accordance with the Directive 2010/63/EU of the European Parliament and Council ‘On the protection of animals used for scientific purposes’. Protocols were approved by the Ethical Committee Charles Darwin N°005 (approval #7475–2016110315516522).

### Experimental animals, parasite and cell lines

We used GFP-expressing *P*. *berghei* (PbGFP, ANKA strain) and *P*. *yoelii* (PyGFP, 17XNL strain) parasite lines, obtained after integration of a GFP expression cassette at the dispensable p230p locus [19]. PbGFP and PyGFP blood stage parasites were propagated in female Swiss mice (6–8 weeks old, from Janvier Labs). *Anopheles stephensi* mosquitoes were fed on PyGFP or PbGFP-infected mice using standard methods [20], and kept at 24°C and 21°C, respectively. PyGFP and PbGFP sporozoites were collected from the salivary glands of infected mosquitoes 14–18 or 21–28 days post-feeding, respectively. HepG2 (ATCC HB-8065), HepG2/CD81 [14] and Hepa1-6 cells (ATCC CRL-1830) were cultured at 37°C under 5% CO2 in DMEM supplemented with 10% fetal calf serum, L-glutamine and antibiotics (Life Technologies), as described [16]. HepG2 and HepG2/CD81 were cultured in culture dishes coated with rat tail collagen I (Becton Dickinson, Le Pont de Claix, France).

### Small interfering RNA silencing of candidate receptors

SiRNA oligonucleotides against EphA2 were designed using E-RNAi (http://www.dkfz.de/signaling/e-rnai3/) and ordered at Eurofins Genomics. Transfection of siRNA oligonucleotides was performed by electroporation in the presence of 10 μL of 20 μM siRNA, as described [14]. Cells were cultured during 48 hours before infection or analysis by immunofluorescence or western blot. After validation of RNAi efficiency by Western blotting, we selected siRNA oligonucleotides targeting mouse EphA2 (5’-GUGCAAGGUGUCCGAUUUU-3’) or human EphA2 (5’-GCACCAAGUGCAUCAAGUA-3’). SiRNA oligonucleotides targeting human CD81 (5’-GCACCAAGUGCAUCAAGUA-3’) or mouse CD81 (5’-CGUGUCACCUUCAACUGUA-3’) were used as a positive control for silencing, as described [14]. As negative controls, we used an irrelevant siRNA oligonucleotide targeting human CD53 (5’-CAACUUCGGAGUGCUCUUC-3’) (siCtrl), or cells electroporated in the absence of siRNA oligonucleotide (mock).

### Western blot

After cell lysis in Tris buffer (Tris 10mM pH 7,4, 150mM NaCl, 1mM CaCL2, 1mM MgCL2) supplemented with 1% NP40 and protease inhibitors (Sigma), soluble fractions were analyzed by Western blot, using a Biorad Mini-Protean® electrophoresis chamber for SDS-PAGE and transfer on PVDF membranes. Membranes were probed with anti-EphA2 D4A2 XP Rabbit mAb (#6997, Cell Signaling Technology) diluted 1/1000, anti-human CD81 (#HPA007234, Sigma Aldrich) diluted 1/2000, or anti-mouse GADPH (#SC25778, Santa Cruz Biotechnology) diluted 1/5000. Chemiluminescence detection was performed using ECL Prime reagents and an ImageQuant LAS 4000 system (GE healthcare Life sciences).

### Immunofluorescence assays

For immunolabeling of EphA2 and CD81, cells were harvested using an enzyme-free Cell Dissociation buffer (Life Technologies), and either directly processed for surface labeling of EphA2 and/or CD81, or treated with the Perm/Fix solution (BD Biosciences) for total labeling of surface and intracellular EphA2. We used the anti-EphA2 rabbit monoclonal antibody D4A2 (#6997, Cell signaling technology, diluted 1/200) and the anti-CD81 mouse monoclonal antibody MT81 (10 μg/ml) [21]. All incubations were performed at 4°C during one hour. After labeling with fluorescent secondary antibodies, cells were analyzed using a Guava EasyCyte 6/2L bench cytometer equipped with a 488 nm and 532 nm lasers (Millipore).

### In vitro infection assays

Cells in 96 well plates (3x10^4^ per well seeded the day before infection) were incubated with 0.5-1x10^4^ PyGFP or PbGFP sporozoites for 3 hours, washed, and further cultured until 24 hours post-infection. Infected cultures were then either trypsinized for detection of GFP-positive cells by flow cytometry, or fixed with 4% paraformaldehyde and analyzed by fluorescence microscopy after labeling with antibodies specific for UIS4 (Sicgen) and the nuclear stain Hoechst 33342 (Life Technologies). For antibody-mediated inhibition assays, we used monoclonal antibodies against EphA2 (rabbit mAb D4A2, Cell Signaling Technology), or human CD81 (1D6, Abcam). A control monoclonal rabbit IgG (DA1E #3900, Cell Signaling Technology) was used as a control. D4A2 was added at 3 dilutions of 10, 5 and 1μg/ml. The DA1E control antibody was added at a dilution equivalent to D4A2 10μg/ml, to control for non-specific effects of azide and glycerol present in D4A2 and DA1E formulations. The anti-CD81 antibody 1D6 was used at 20μg/ml.

### Statistical analysis

Statistical significance was assessed by non-parametric analysis using Kruskal-Wallis and Mann-Whitney tests. Correlation between EphA2 and CD81 expression levels was analyzed using Pearson correlation. All statistical tests were computed with GraphPad Prism 7. Significance was defined as p<0.05. *In vitro* experiments were performed at least three times, with a minimum of three technical replicates per experiment.

## Results

### EphA2 expression in hepatocytic cell lines

**Fig 1:**
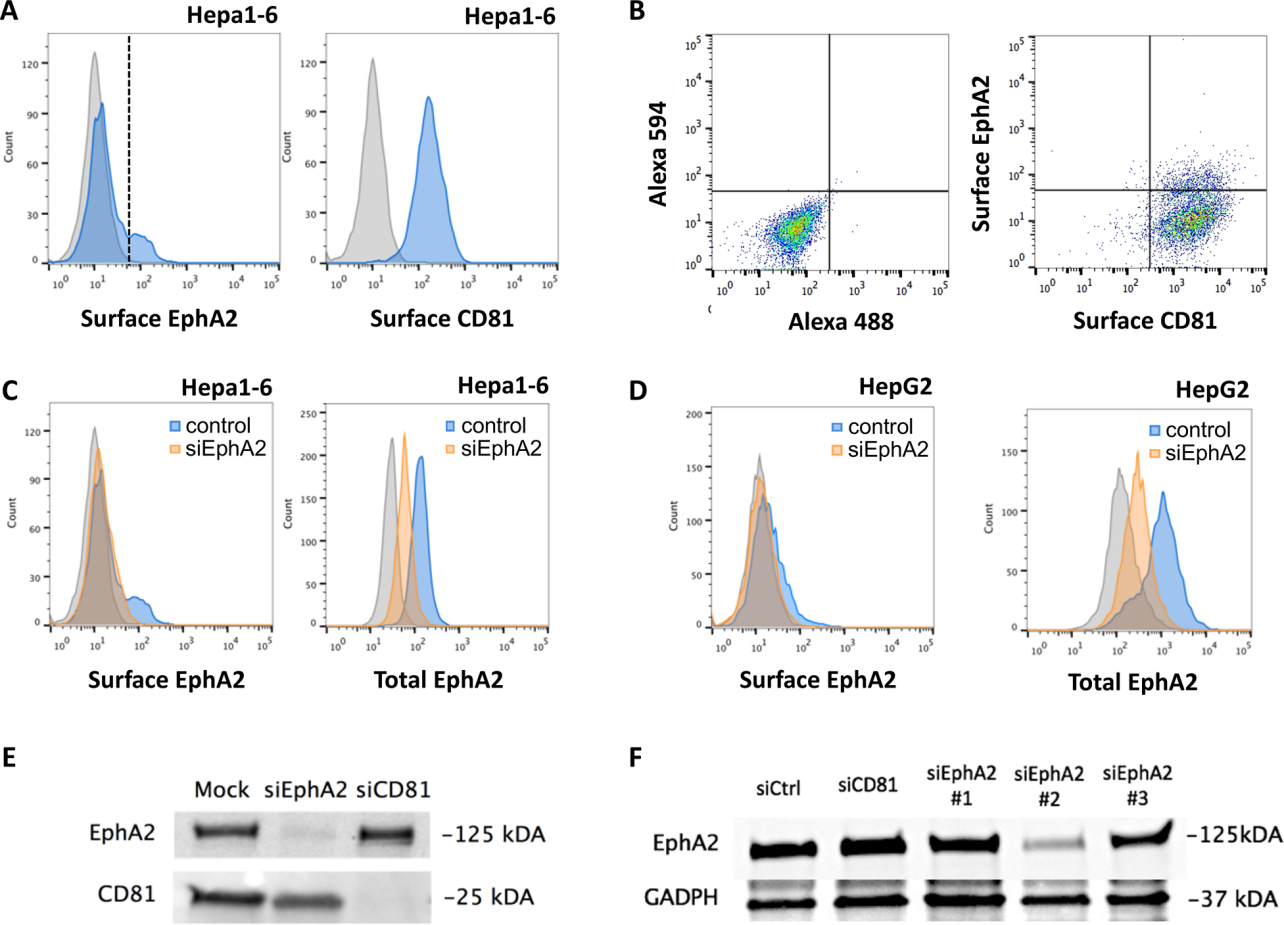
EphA2 receptor expression in hepatocytic cell lines. **A.** Hepa1-6 cells were stained with anti-EphA2 D4A2 (blue, left panel) or anti-CD81 MT81 (blue, right panel) followed by Alexa Fluor-488 conjugated secondary antibodies, and analyzed by FACS. The grey histograms represent cells stained with secondary antibody only (negative control). The vertical line represents the threshold used to distinguish EphA2^low^ and EphA2^high^ cells **B.** Hepa1-6 cells were stained with anti-EphA2 D4A2 (rabbit) and anti-CD81 MT81 (rat) primary antibodies, followed by Alexa Fluor-594 (anti-rabbit) and Alexa Fluor-488 (anti-rat) secondary antibodies, and analyzed by FACS (dot plot on the left). As a control, cells were stained with secondary antibodies only (dot plot on the left). There was no correlation between EphA2 and CD81 fluorescence intensities (Pearson coefficient R^2^ = 0,263). **C.** Hepa1-6 cells were transfected with siRNA oligonucleotides targeting mouse EphA2. 48 hours after transfection, surface (left panel) or total (right panel) EphA2 expression was analyzed by FACS in control (blue) and siEphA2-treated (orange) cells. The grey histograms correspond to controls cells stained with secondary antibodies only. **D.** HepG2 cells were transfected with siRNA oligonucleotides targeting human EphA2. 48 hours after transfection, surface (left panel) or total (right panel) EphA2 expression was analyzed by FACS in control (blue) and siEphA2-treated (orange) cells. The grey histograms correspond to controls cells stained with secondary antibodies only. **E.** HepG2/CD81 cells were transfected with siRNA oligonucleotides targeting human EphA2 or CD81, or electroporated without siRNA (mock). Cells were lysed 48 hours post-transfection and protein extracts were analyzed by Western blot, using antibodies specific for EphA2 or CD81. **F**. Hepa1-6 cells were transfected with siRNA oligonucleotides targeting mouse EphA2 or CD81, or with a control siRNA (siCtrl). Cells were lysed 48 hours post-transfection and protein extracts were analyzed by Western blot, using antibodies specific for EphA2 or GAPDH. Among three siRNA oligonucleotides tested against mouse EphA2, siRNA #2 was the most efficient and was used throughout the study.

We first analyzed EphA2 expression in Hepa1-6 cells using immunofluorescence labeling and flow cytometry. Hepa1-6 is a mouse hepatoma cell line that supports *P*. *berghei* and *P*. *yoelii* infection. Both parasites use a CD81-dependent pathway in this model as Hepa1-6 cells express CD81 but not SR-B1 [15]. A bimodal distribution of EphA2 surface expression was observed on Hepa1-6 cells by FACS, with one population where EphA2 signal was not distinguishable from the background, and another one expressing higher levels of EphA2 (Fig 1A). These two populations are hereafter referred to as EphA2^low^ and EphA2^high^ cells, respectively. These results are consistent with the previous observations from Kaushansky *et al*. showing an heterogeneous distribution of EphA2 receptor among hepatoma cells [17]. Interestingly, we observed an homogeneous distribution of EphA2 after cell permeabilization (Fig 1C, total EphA2), suggesting that a major pool of EphA2 is localized intracellularly. We then performed double labeling of EphA2 and CD81 to analyze whether EphA2 surface expression correlates with CD81 expression (Fig 1B). We did not observe any correlation between EphA2 and CD81 surface expression levels (Pearson coefficient R^2^ = 0,263), indicating that the higher susceptibility of EphA2^high^ cells reported by Kaushanksy *et al*. is not linked to higher expression of CD81, which is essential for both *P*. *yoelii* and *P*. *berghei* sporozoite infection in this cellular model [14,21].

In contrast with Hepa1-6 cells, the human hepatocarcinoma HepG2 cells express SR-BI but not CD81, and as a consequence support infection by *P*. *berghei* but not *P*. *yoelii* sporozoites [14,15]. Ectopic expression of CD81 in genetically engineered HepG2/CD81 cells is sufficient to render these cells susceptible to *P*. *yoelii* infection, and provides *P*. *berghei* sporozoites with an alternative route for entry [14,15]. FACS analysis revealed a HepG2 cells express very low level of EphA2 on their surface (Fig 1D, surface EphA2). The signal was more intense when cells were permeabilized before labeling with antibodies (Fig 1D, total EphA2), suggesting that a large pool of EphA2 is intracellular in HepG2 cells, as observed with murine Hepa1-6 cells.

In order to confirm the specificity of EphA2 staining, we used small interfering RNA (siRNA) oligonucleotides to inhibit EphA2 gene expression. We designed siRNAs for human and mouse EphA2 and transfected them into HepG2, HepG2/CD81 or Hepa1-6 cells. As controls, cells were transfected either with an irrelevant siRNA oligonucleotide targeting human CD53 (siCtrl) or with no siRNA (mock). Flow cytometry analysis 48 hours after siRNA transfection revealed a marked reduction of the mean fluorescence intensity in the silenced cell population compared to the control population in both permeabilized and non-permeabilized conditions, reflecting the efficient depletion of both extracellular and intracellular receptor pools in Hepa1-6 (Fig 1C) and HepG2 cells (Fig 1D). Western blotting confirmed that EphA2 protein levels are reduced by 75–85% with the most efficient siRNAs in Hepa1-6 and HepG2/CD81 cells, respectively (Fig 1E and 1F).

### EphA2 is not required for *P*. *yoelii* sporozoite infection

**Fig 2:**
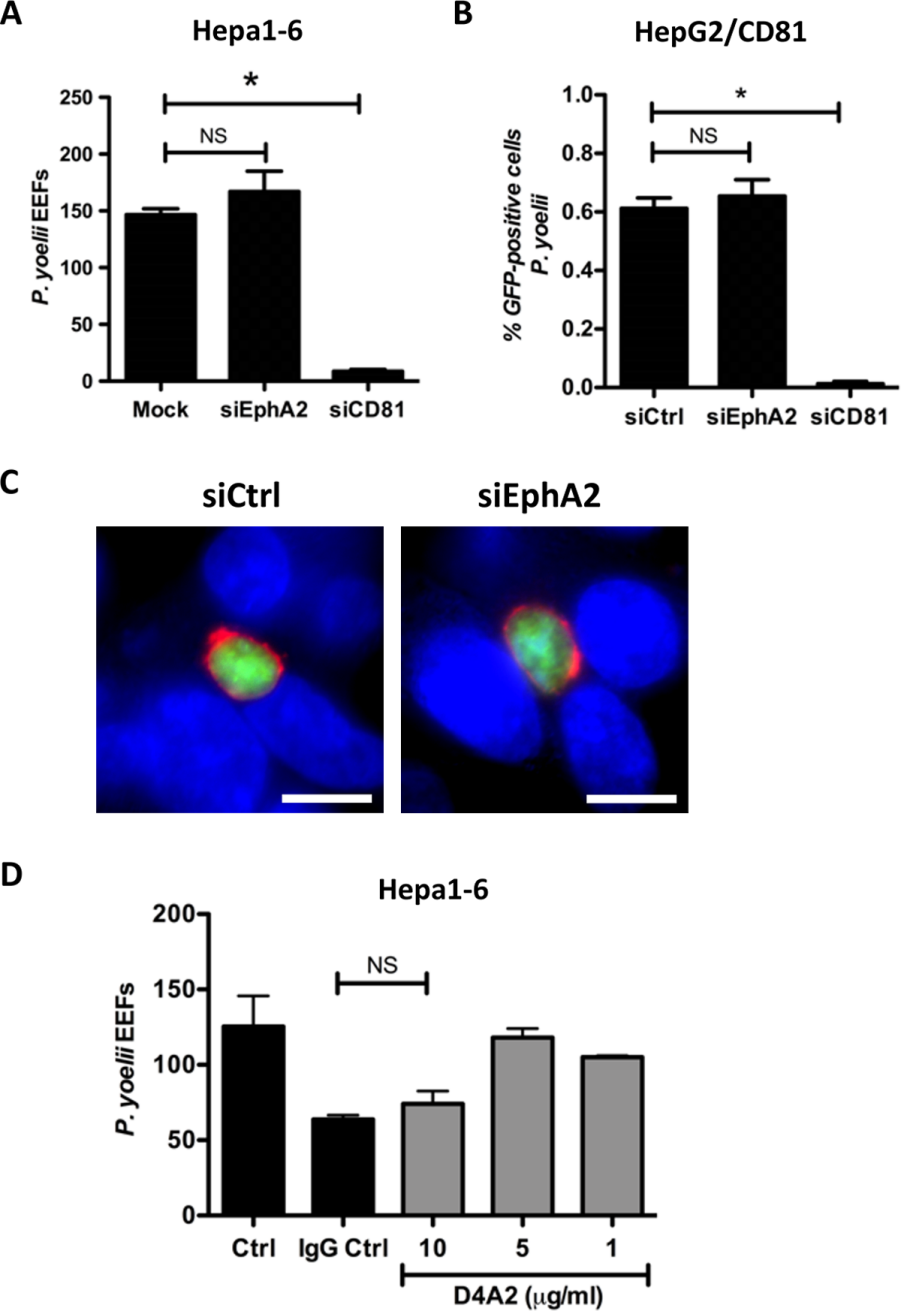
EphA2 inhibition does not impair *P*. *yoelii* infection. **A.** Hepa1-6 cells were transfected with siRNA oligonucleotides targeting mouse EphA2 or CD81, or electroporated without siRNA (mock). Cells were infected with *PyGFP* sporozoites 48 hours after siRNA transfection, and the number of EEFs 24 hours post-infection was determined by fluorescence microscopy after UIS4 staining. NS, non-significant; *, p<0.05 (Kruskal-Wallis with Dunns post-test). **B.** HepG2/CD81cells were transfected with siRNA oligonucleotides targeting human EphA2 or human CD81, or with a control siRNA (siCtrl). Cells were infected with *PyGFP* sporozoites 48 hours after siRNA transfection, and the percentage of infected (GFP-positive) cells was determined by FACS 24 hours post-infection. NS, non-significant; *, p<0.05 (Kruskal-Wallis with Dunns post-test). **C.** HepG2/CD81 cells were transfected with siRNA targeting EphA2 or with a control siRNA (siCtrl) and infected 48 hours later with *PyGFP* sporozoites. Infected cultures were fixed 24 hours post-infection and stained with anti-UIS4 antibodies (red) and the nuclear stain Hoechst 33342 (blue). The images show PyGFP EEFs (green) surrounded by a UIS4-positive PVM (red). Scale bar, 10 μm. **D.** Hepa1-6 cells were incubated with PyGFP sporozoites in the presence of increasing concentrations of the EphA2-blocking antibody D4A2, of a control antibody (DA1E, IgG Ctrl, used at the same dilution as D4A2 highest concentration) or without antibody (Ctrl). The number of EEFs was determined 24 hours post-infection by fluorescence microscopy after UIS4 staining. NS, non-significant (Kruskal-Wallis with Dunns post-test).

We next measured the impact of EphA2 silencing on CD81-dependent infection using GFP-expressing *P*. *yoelii* parasites (PyGFP) [15]. *P*. *yoelii* sporozoites strictly require CD81 for infection, and can invade Hepa1-6 cells and HepG2/CD81 cells, but not the parental HepG2 cells that do not express CD81 [14]. Hepa1-6 cells were transfected with a siRNA against mouse EphA2 (validated siRNA#2), and incubated 48 hours after transfection with PyGFP sporozoites. Infection rates were then measured at 24 hours post-infection by immunostaining of the PVM marker UIS4 and microscopy counting of EEFs. Silencing of EphA2 in Hepa1-6 cells had no significant effect on PyGFP infection as compared to the control condition, whereas at the opposite silencing of CD81 abrogated infection, as expected (Fig 2A) [14]. In order to confirm these findings in another cell type, we analyzed the effects of EphA2 silencing in HepG2/CD81 cells, using flow cytometry to quantify PyGFP-infected cells 24 hours post-infection (Fig 2B). The HepG2/CD81 infection rate was not altered after siEphA2, as compared to the control, but was dramatically reduced after siCD81, as expected. Moreover, UIS4 staining revealed no defect in PVM formation after EphA2 silencing (Fig 2C).

As an alternative approach to neutralize EphA2, we used a rabbit monoclonal antibody (D4A2) that recognize both human and mouse EphA2, and was previously shown to reduce sporozoite infection *in vitro* [17]. Because available D4A2 formulations contain azide and glycerol, we used a control rabbit IgG (DA1E) prepared in the same conditions as D4A2 by the manufacturer. Hepa1-6 cells were incubated with PyGFP in the presence of anti-EphA2 or control antibody, and EEFs were counted at 24 hours post-invasion by microscopy, after immunostaining of UIS4. As shown in Fig 2D, a partial inhibition was observed only at the highest concentration of D4A2 (10 μg/ml). However, a similar inhibition was observed with the control IgG, strongly suggesting that this is a non-specific effect due to the presence of sodium azide and/or glycerol in the antibody formulations.

### EphA2 is not required for *P*. *berghei* sporozoite infection

**Fig 3:**
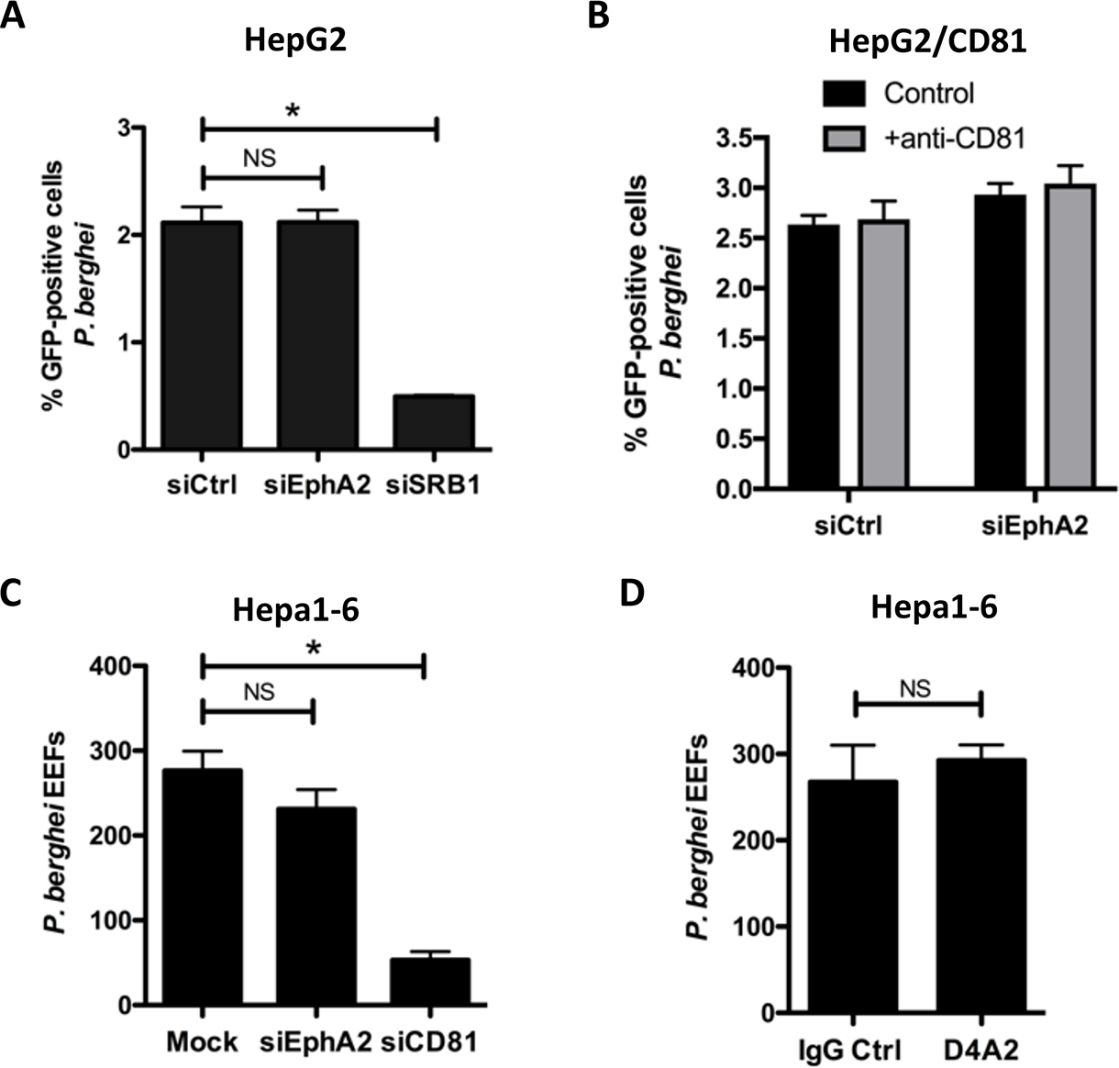
EphA2 is not required for *P*. *berghei* infection. **A.** HepG2 cells were transfected with siRNA oligonucleotides targeting human EphA2 or SR-BI, or with a control siRNA (siCtrl). Cells were infected with *PbGFP* sporozoites 48 hours after siRNA transfection, and the percentage of infected (GFP-positive) cells was determined by FACS 24 hours post-infection. NS, non-significant; *, p<0.05 (Kruskal-Wallis with Dunns post-test). **B.** HepG2/CD81 cells were transfected with siRNA oligonucleotides targeting human EphA2 or with a control siRNA (siCtrl). Cells were infected with *PbGFP* sporozoites 48 hours after siRNA transfection, in the absence or presence of a CD81-blocking antibody (1D6, 20 μg/ml) and the percentage of infected (GFP-positive) cells was determined by FACS 24 hours post-infection. **C.** Hepa1-6 cells were transfected with siRNA oligonucleotides targeting mouse EphA2 or CD81, or electroporated without siRNA (mock). Cells were infected with *PbGFP* sporozoites 48 hours after siRNA transfection, and the number of EEFs 24 hours post-infection was determined by fluorescence microscopy after UIS4 staining. NS, non-significant; *, p<0.05 (Kruskal-Wallis with Dunns post-test). **D.** Hepa1-6 cells were incubated with PbGFP sporozoites in the presence of the EphA2-blocking antibody D4A2 (5 μg/ml) or a control antibody (IgG Ctrl), and the number of EEFs 24 hours post-infection was determined by fluorescence microscopy. NS, non-significant (Mann-Whitney U Test, two-tailed).

Unlike *P*. *yoelii*, *P*. *berghei* sporozoites can use SR-BI to invade cells through an alternative CD81-independent entry pathway, including in HepG2 cells [15]. To analyze whether EphA2 receptor could contribute to CD81-independent entry, we silenced EphA2 with siRNA oligonucleotides in HepG2 cells, followed by incubation with PbGFP sporozoites 48 hours later. Quantification of infection by FACS 24 hours post-infection revealed no significant difference between the control and siEphA2-treated cells (Fig 3A). On the other hand, silencing of SR-BI drastically reduced infection, as expected [15]. A similar result was obtained in HepG2/CD81 cells upon blocking of the CD81 pathway. In HepG2/CD81 cells, *P*. *berghei* sporozoites can use SR-BI or CD81, alternatively, for invasion. The silencing of EphA2 with siRNA had no effect on PbGFP infection of HepG2/CD81 cells, both in the presence or in the absence of a CD81 neutralizing antibody (Fig 3B). Finally, inhibition of mouse EphA2 using siRNA or antibodies had no significant effect on *P*. *berghei* infection in Hepa1-6 cells (Fig 3C and 3D). Altogether, these data do not support a role for the EphA2 receptor in hepatocyte infection by *P*. *berghei*, irrespective of the entry pathway.

### Correlation between EphA2 expression level and infection

**Fig 4:**
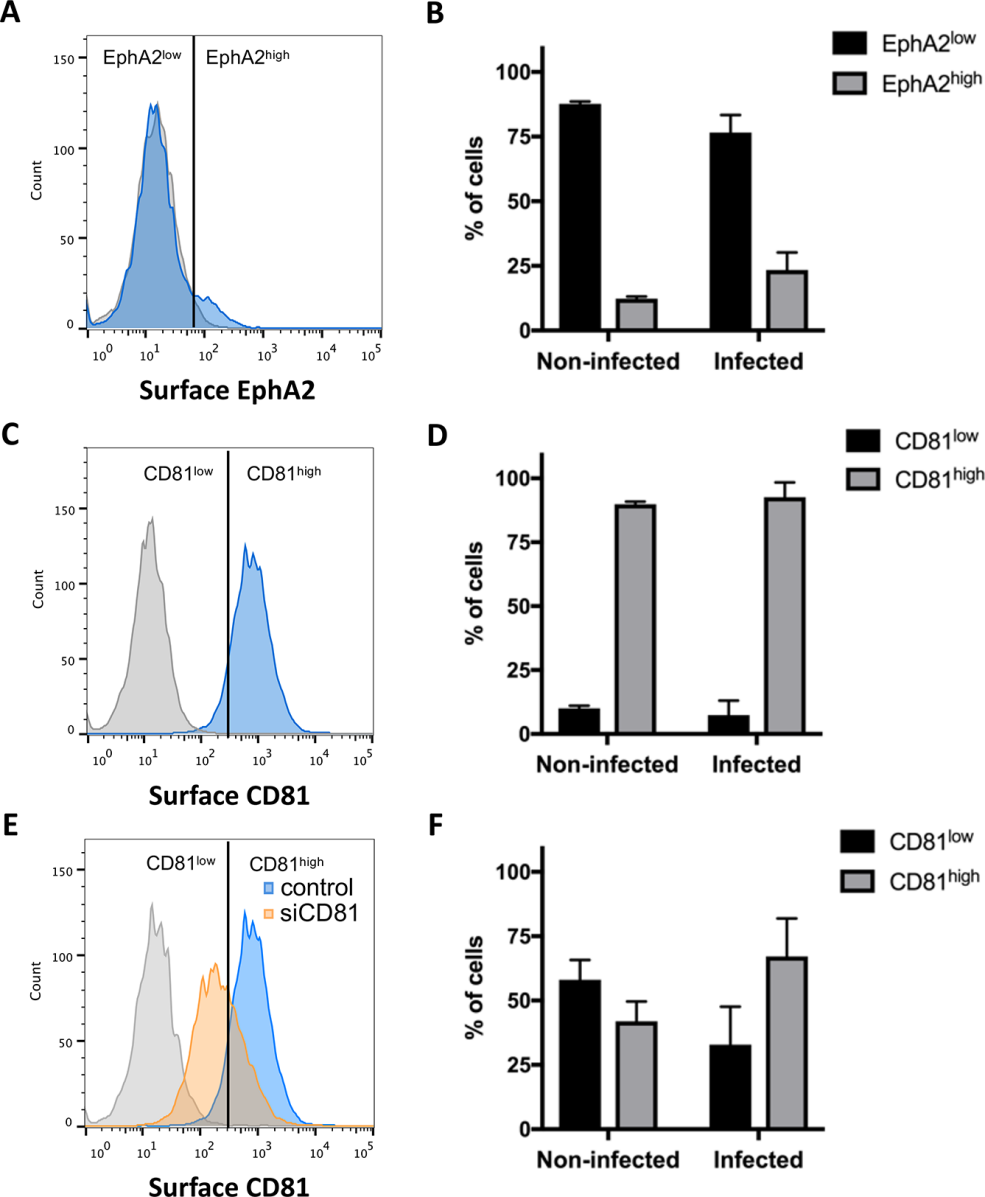
Correlation between EphA2 expression level and infection rates. **A.** Hepa 1–6 cells infected with PbGFP sporozoites were stained 24 hours post-infection with an anti-EphA2 primary antibody (D4A2) followed by Alexa Fluor 594 secondary antibodies (blue histogram), and analyzed by FACS. The grey histogram corresponds to control cells labeled with secondary antibodies only. The vertical line indicates the threshold used to distinguish EphA2^low^ and EphA2^high^ cells. **B.** The percentage of EphA2^low^ and EphA2^high^ cells, was determined in non-infected (GFP-negative) and infected (GFP-positive) cells. **C.** Hepa 1–6 cells infected with PbGFP sporozoites were stained 24 hours post-infection with an anti-CD81 primary antibody (MT81) followed by Alexa Fluor 594 secondary antibodies (blue histogram), and analyzed by FACS. The grey histogram corresponds to control cells labeled with secondary antibodies only. The vertical line indicates the threshold used to distinguish CD81^low^ and CD81^high^ cells. This threshold was arbitrarily defined to obtain a majority of CD81^low^ population after siRNA silencing of CD81. **D.** The percentage of CD81^low^ and CD81^high^ cells, was determined in non-infected (GFP-negative) and infected (GFP-positive) cells. **E.** Hepa1-6 cells were transfected with siRNA targeting CD81 and infected with PbGFP sporozoites. Cells were stained 24 hours post-infection with an anti-CD81 primary antibody (MT81) followed by Alexa Fluor 594 secondary antibodies (orange histogram), and analyzed by FACS. The grey histogram corresponds to control cells labeled with secondary antibodies only, and the blue histogram corresponds to the control cells labeled with CD81 antibodies, as in C. The vertical line indicates the same threshold as in C, used to distinguish CD81^low^ and CD81^high^ cells. **F.** The percentage of CD81^low^ and CD81^high^ cells was determined in non-infected (GFP-negative) and infected (GFP-positive) CD81 siRNA-treated Hepa1-6 cells.

We next investigated whether EphA2, despite being dispensable, could be a marker associated with host cell susceptibility, as proposed by Kaushansky *et al*. [17]. We analyzed whether EphA2 surface expression correlates with parasite infection rates, using the Hepa1-6 cell model and PbGFP sporozoites. Following infection with PbGFP sporozoites, cells were recovered at 24 hours post-infection, labeled with anti-EphA2 or anti-CD81-antibodies and analyzed by FACS. As observed with uninfected cell cultures (Fig 1), most non-infected cells from infected cultures were EphA2^low^, and only a minor population were EphA2^high^ (Fig 4A). A similar distribution was observed among infected cells (Fig 4B). In contrast with EphA2, CD81 was expressed uniformly on the surface of cells from infected Hepa1-6 cultures (Fig 4C), and, as expected, both uninfected and infected cells were mostly CD81^high^ (Fig 4D). Interestingly, when the same analysis was done after CD81 silencing, to increase the proportion of CD81^low^ cells (Fig 4E), there was still a majority of infected cells that were CD81^high^ (Fig 4F). These results show that *P*. *berghei* sporozoites preferentially infect cells with a high CD81 expression at their surface, irrespective of the EphA2 expression level.

### EphA2 is not required for sporozoite cell traversal

**Fig 5:**
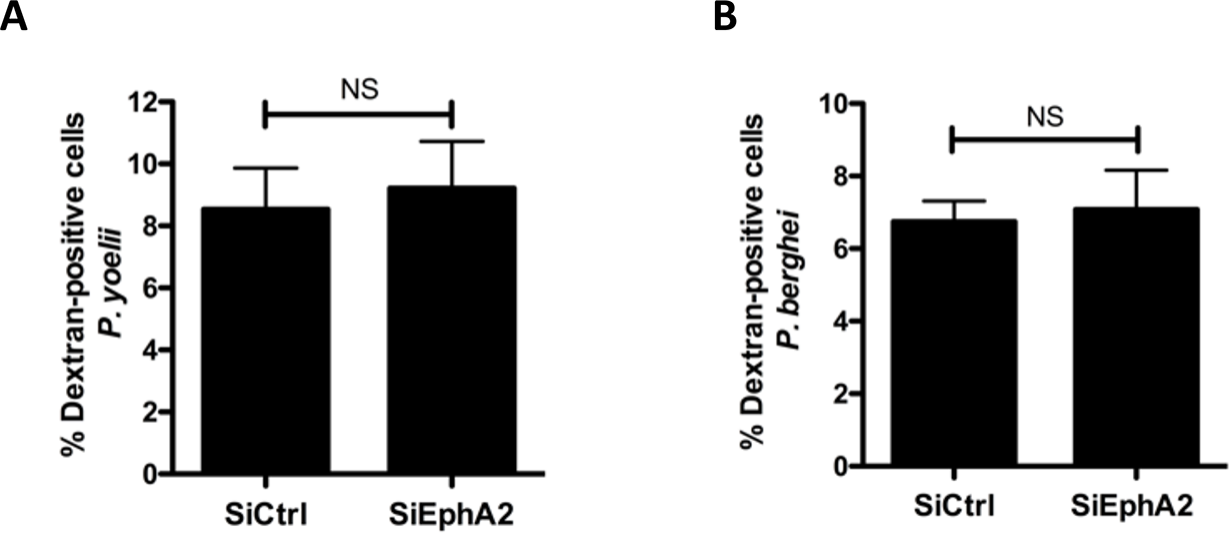
EphA2 is not required for sporozoite cell traversal. **A-B**. Hepa1-6 cells were transfected with siRNA oligonucleotides targeting mouse EphA2 (siEphA2) or with a control siRNA (siCtrl). 48 hours after siRNA transfection, cells were incubated for 1.5 hours with PyGFP (**A**) or PbGFP (**B**) sporozoites in the presence of rhodamine-labeled dextran, and the number of traversed (dextran-positive) cells was determined by FACS. NS, non-significant (Mann-Whitney U Test, two-tailed).

In their study, Kaushansky *et al*. used antibodies against the circumsporozoite protein (CSP) to assess *P*. *yoelii* infection rates by flow cytometry [17]. CSP detection may not discriminate between traversed cells and productively infected cells, as CSP is shed upon parasite gliding and cell traversal [22]. Since the proportion of traversed cells usually exceeds that of productively invaded cells, we reasoned that the correlation observed between EphA2 levels and infection rates could be due to an effect on cell traversal rather than infection. We analyzed the traversal activity of *P*. *berghei* or *P*. *yoelii* sporozoites in EphA2-silenced versus control cell cultures, using flow cytometry to measure the uptake of fluorescent rhodamine-dextran tracer by wounded traversed cells [23,24]. There was no significant difference in dextran uptake between these two conditions, for both parasites (Fig 5A and 5B), indicating that EphA2 silencing does not impact sporozoite cell traversal activity. Collectively, our data thus do not support any implication of EphA2 receptor in *Plasmodium* sporozoite cell traversal or productive invasion.

## Discussion

Sporozoite invasion of hepatocytes is a potential target for malaria vaccines, yet the underlying molecular mechanisms still remain poorly characterized. Determining the role of host cell receptors may help to discover new parasite ligands and also to understand the signaling cascades leading to productive infection. In this study, we revisited the role of host EphA2 receptor during infection of hepatocytic cells by two rodent malaria parasites, *P*. *yoelii* and *P*. *berghei*. We performed siRNA and antibody-based functional assays, using GFP-expressing parasites for accurate detection of infected cells by flow cytometry or microscopy. In good agreement with previous observations [17], we observed some heterogeneity in EphA2 expression level among cells in murine Hepa1-6 cells. However, in our hands, there was no clear correlation between the infection rate and EphA2 expression level. Most importantly, inhibition of EphA2 by siRNA or antibodies had no effect on *P*. *yoelii* and *P*. *berghei* sporozoite infection. The number of infected cells, the formation of a UIS4-positive PVs and the morphology of the developing EEFs were not altered upon EphA2 silencing. Moreover, sporozoite cell traversal activity was not affected by EphA2 depletion. These results argue against an essential role of EphA2 during malaria liver infection.

EphA2 has been previously identified as a host entry factor for HCV, acting downstream of SR-BI and CD81 [18]. Differently from HCV, CD81 and SR-BI play independent roles during *Plasmodium* infection [15]. Whilst *P*. *falciparum*, like *P*. *yoelii*, relies on CD81 for infection, *P*. *vivax* sporozoites enter hepatocytes *via* a SR-BI-dependent pathway. *P*. *berghei*, on the other hand, can use alternatively CD81-dependent or SR-BI-dependent entry routes, depending on the host cell type [15]. Here, we specifically explored the contribution of EphA2 in both CD81- and SR-BI-associated pathways by exploiting *P*. *yoelii* and *P*. *berghei* models and different cellular systems. Our data reveal that EphA2 does not play any important role irrespective of the sporozoite entry route.

The discrepancy between our results and the study of Kaushansky *et al*. [17] might be explained by differences in the experimental procedures. In our study, transgenic parasites expressing GFP were used to measure accurately cell infection by flow cytometry or microscopy, whereas Kaushansky *et al*. relied mainly on CSP labeling to quantify invasion. As CSP is shed from migrating sporozoites, CSP-based analysis may not discriminate accurately productive invasion and cell traversal, especially at early time points (90 min) as performed in the Kaushansky *et al*. study. In this regard, we have shown that sporozoites commit to productive invasion after an extended period (30–90 min) of cell traversal [25]. Here, we measured infection rates at 24 hours after adding sporozoites, and used the PVM marker UIS4, to specifically analyze productive invasion. Our data show that depletion or blocking of EphA2 has no impact on either the number of infected cells or the formation of UIS4-labeled PVs. Interestingly, a reduction of *P*. *yoelii* liver stage burden was observed in *Epha2*^*(-/-)*^ mice, associated with a delayed onset of blood stage infection [17]. Although one cannot exclude that EphA2 participates in sporozoite invasion *in vivo*, other phenotypic defects caused by the absence of EphA2 could also contribute, directly or indirectly, to the lower susceptibility of *Epha2*^*(-/-)*^ mice. Importantly, in contrast to *Cd81*^*(-/-)*^ mice, which are refractory to *P*. *yoelii* sporozoite infection [13], *Epha2*^*(-/-)*^ mice remain susceptible [17], confirming that EphA2 is dispensable for liver infection.

Kaushansky *et al*. proposed that EphA2 acts as a putative receptor for the sporozoite protein P36, a member of the *Plasmodium* 6-cysteine domain (6-cys) protein family. NMR structural analysis of one 6-cys domains of *P*. *falciparum* P12 protein revealed a beta sandwich fold with homology to ephrins [26]. Two 6-cys proteins expressed in sporozoites, P36 and P52, are essential for liver infection [27–30]. Our recent study using genetic complementation in *P*. *berghei* and *P*. *yoelii* revealed that both proteins are required for sporozoite productive invasion, and identified P36 as a key parasite determinant of the host cell entry pathways used by sporozoites for infection [15]. In their study, Kaushansky *et al*. reported a reduced correlation between EphA2 expression level and host cell invasion by P36/P52-depleted parasites [17]. However, a bias was still observed toward EphA2^High^ cells among cells harboring mutant parasites. As *p36/p52*-knockout sporozoites do not productively invade cells [15], these results argue against a functional interaction between EphA2 and P36 (or P52) for host cell infection. Nevertheless, a recombinant form of P36 was shown to interfere with EphA2 phosphorylation mediated by its ligand Ephrin A1 [17]. This observation raises the possibility that P36 affects EphA2 signaling indirectly, possibly by acting on other host cell receptors, including SR-BI or CD81.

In conclusion, our data do not support any important role for EphA2 during *Plasmodium* sporozoite host cell invasion, irrespective of the entry route. Although dispensable for sporozoite entry, EphA2 receptor could be implicated in a post-invasion signaling cascade triggered upon invasion. Further studies are needed to elucidate the function of CD81 and SR-BI during infection and to unravel the molecular interactions leading to parasite host cell invasion in the liver.

## Supporting information

S1 Appendix *Plasmodium* EphA2 raw data set. This is the Excel file with the raw data (xlsx).
(XLSX)
